# Euglycaemic ketoacidosis associated with dual-acting GLP-1/GIP receptor agonist treatment following bariatric surgery

**DOI:** 10.1530/EDM-26-0059

**Published:** 2026-09-21

**Authors:** Deirdre Green, Nigel Glynn

**Affiliations:** Department of Endocrinology, Mater Misericordiae University Hospital, Dublin, Ireland

**Keywords:** obesity, metabolism

## Abstract

**Summary:**

Euglycaemic ketoacidosis is characterised by high anion gap metabolic acidosis with elevated serum ketone bodies in the absence of significant hyperglycaemia. Although traditionally associated with sodium-glucose cotransporter 2 (SGLT-2) inhibitors, incretin-based therapies have recently been implicated. Tirzepatide is a dual-acting glucagon-like peptide 1 (GLP-1)/glucose-dependent insulinotropic polypeptide (GIP) receptor agonist. It is an approved treatment for obesity in patients without diabetes mellitus. We present the case of a 51-year-old woman without diabetes, 2 years after sleeve gastrectomy, who presented with nausea, vomiting and abdominal pain after rapid dose escalation of tirzepatide. Laboratory evaluation revealed severe metabolic acidosis (pH 7.01, venous bicarbonate 9.2 mmol/L), elevated serum ketones (6 mmol/L) and normal blood glucose (5 mmol/L), consistent with euglycaemic ketoacidosis. Plasma lactate was normal (1.4 mmol/L). She was managed with intravenous fluids, insulin infusion and anti-emetics until the symptoms and acidosis resolved. Tirzepatide was discontinued. This case highlights the potential for dual-acting GLP-1/GIP receptor agonists to increase susceptibility to euglycaemic ketoacidosis in patients without diabetes, particularly in the context of prior bariatric surgery. Clinicians should consider euglycaemic ketoacidosis in patients receiving dual-acting GLP-1/GIP therapy who present unwell with metabolic acidosis.

**Learning points:**

- Euglycaemic ketoacidosis with GLP-1/GIP receptor agonist use following bariatric surgery is rarely reported.
- Clinicians should consider ketoacidosis in patients on GLP-1/GIP receptor agonist therapy who have unexplained metabolic acidosis.
- Measuring urine and/or serum ketone levels is necessary in patients presenting with gastrointestinal symptoms who are taking dual-acting GLP-1/GIP receptor agonists.

## Background

Ketoacidosis, characterised by the presence of elevated ketoacids (acetoacetate and beta-hydroxybutyrate) and high anion gap metabolic acidosis, is a life-threatening condition. The liver produces ketoacids through the breakdown of free fatty acids, which are metabolised by extrahepatic tissues as an energy source. This conversion to ketoacids is commonly observed in patients with diabetes due to insulin deficiency (1). Euglycaemic ketoacidosis is traditionally associated with sodium-glucose cotransporter 2 (SGLT-2) inhibitor use. However, there are less data on the risk associated with glucagon-like peptide 1 (GLP-1) and dual-acting GLP-1 and glucose-dependent insulinotropic polypeptide (GIP) receptor agonists.

Tirzepatide is a dual-acting GLP-1/GIP receptor agonist administered as a once-weekly subcutaneous injection. It has recently been approved for the treatment of obesity in adults with and without diabetes mellitus. We present a case of euglycaemic ketoacidosis in a patient without diabetes who had previously had bariatric surgery.

## Case presentation

A 51-year-old woman with a body mass index (BMI) of 24 kg/m^2^ presented to the emergency department with a three-day history of nausea, vomiting and abdominal pain. Her medical history included hepatic steatosis, obstructive sleep apnoea, hypothyroidism, hypertension, migraine and depression. Her medications included tirzepatide 15 mg subcutaneously weekly, amitriptyline, bisoprolol, levothyroxine, pantoprazole, pregabalin, sertraline, venlafaxine and candesartan. She had no history of diabetes mellitus. She had sleeve gastrectomy performed 2 years previously. Her BMI prior to bariatric surgery was 38.9 kg/m^2^.

Eight weeks prior to presentation, she commenced tirzepatide 2.5 mg subcutaneously once weekly. The dosage was rapidly escalated to 15 mg once weekly over this 8-week period. She had previously taken tirzepatide for 3 months but discontinued the medication after presenting to an emergency department overseas with similar symptoms. She remained off tirzepatide for 1 month before recommencing therapy on this occasion. Her weight prior to starting tirzepatide was 74 kg (BMI: 28.2 kg/m^2^). At presentation to our hospital, she weighed 63 kg (BMI: 24 kg/m^2^).

On presentation, she was dehydrated with a heart rate of 128 beats per minute and blood pressure of 144/75 mmHg. Physical examination revealed epigastric tenderness but was otherwise unremarkable.

## Investigations

The laboratory results are shown in Table 1*.* Blood gas analysis revealed a pH of 7.01 (reference range: 7.35–7.45), a partial pressure of carbon dioxide of 4.51 kPa (reference range: 4.5–6.0) and a bicarbonate concentration of 9.2 mmol/L (reference range: 22.4–25.8). The anion gap was 14 (reference range: <12). Serum ketone concentration was 6 mmol/L (reference range: <0.2 mmol/L). Blood glucose was normal (5 mmol/L). Plasma lactate was also normal (1.4 mmol/L). Glycosylated haemoglobin (HbA1c) was 32 mmol/mol (reference range: 20–42 mmol/mol).

**Table 1:** Laboratory results at presentation.

Item	Value	Reference range
WBC (×10^9^/L)	16.95	3.5–11
Haemoglobin (g/dL)	16.7	11.5–16.5
Platelet count (×10^9^/L)	575	150–400
Sodium (mmol/L)	133	133–146
Serum potassium (mmol/L)	3.6	3.3–5.0
Blood urea nitrogen (mmol/L)	4.7	2.8–8.6
Corrected calcium (mmol/L)	2.61	2.2–2.6
Phosphate (mmol/L)	0.66	0.74–1.52
Magnesium (mmol/L)	0.74	0.7–1.0
Total bilirubin (μmol/L)	4	5–24
GGT (IU/L)	25	8–53
AST (IU/L)	9	0–55
Creatinine (μmol/L)	48	46–86
Glucose (mmol/L)	5	
HbA1c (mmol/mol)	32	20–42
Amylase (IU/L)	75	28–97
C-reactive protein (mg/L)	11	<7
Lactate* (mmol/L)	1.4	0.5–2.0
Paracetamol level (mg/L)	15	
Salicylates (mg/L)	<50	

* Performed using a point-of-care analyser.

WBC, white blood cells; AST, aspartate aminotransferase; GGT, gamma-glutamyl transpeptidase; HbA1c, glycosylated haemoglobin.

## Treatment

The patient was admitted to the high dependency unit for 72 h for the management of high anion gap euglycaemic ketoacidosis. She was fluid resuscitated with 0.9% saline. She was commenced on a variable rate insulin infusion alongside intravenous 5% dextrose. The insulin infusion was initiated at 0.5 units/h and titrated between 0.5 and 2 units/h according to blood glucose levels. Intravenous insulin was required for approximately 48 h. Figure 1 outlines the trend in pH, blood ketone and bicarbonate concentration. Regular anti-emetics were prescribed.

**Figure 1:**
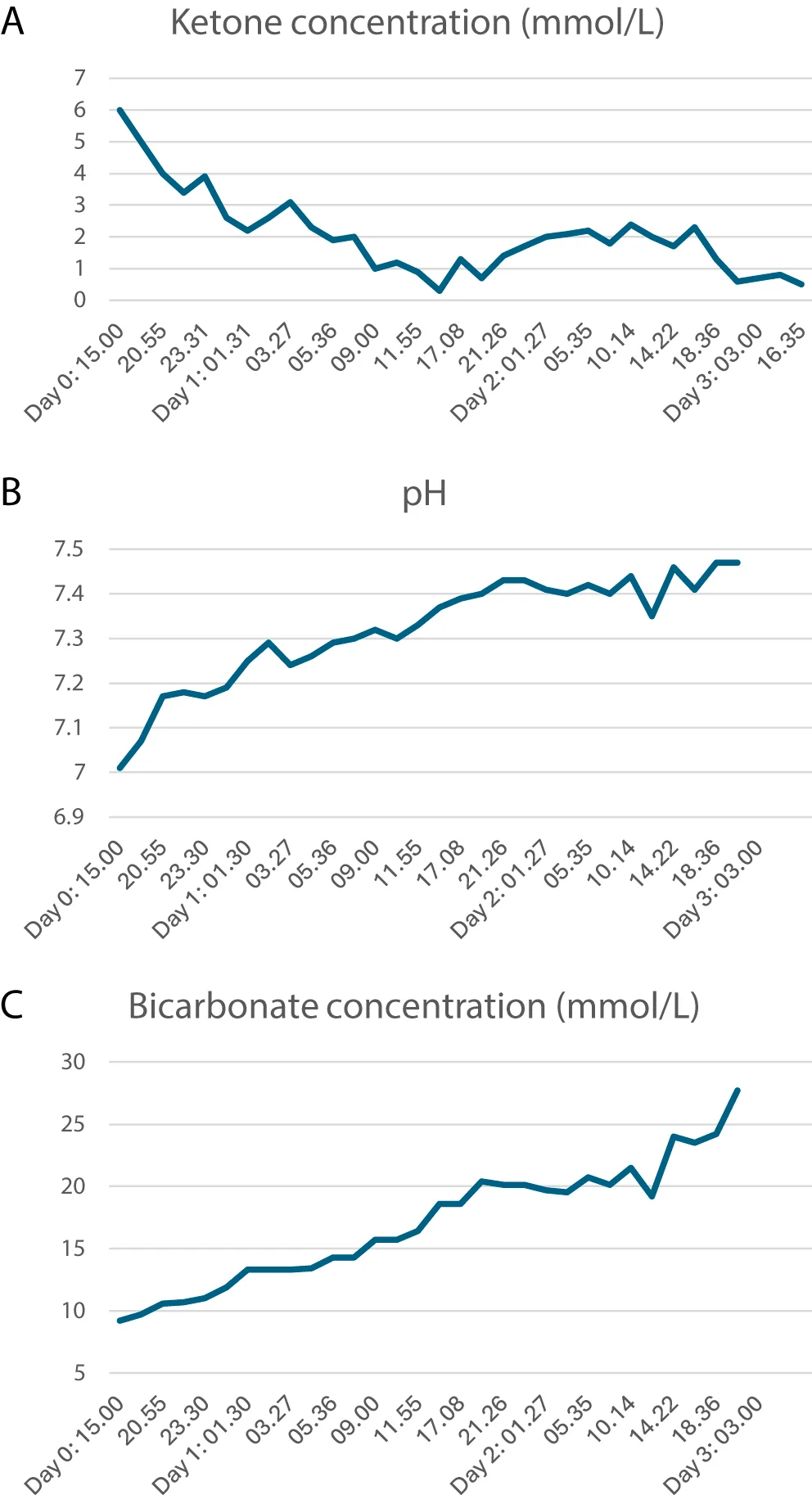
Ketone, pH and bicarbonate trend.

## Outcome and follow-up

The anion gap resolved, acidosis was corrected, and the patient was advised against tirzepatide use. She was discharged from hospital after four days.

## Discussion

GLP-1 and GIP are gut-derived incretin hormones. They are released in response to ingestion of carbohydrates and fats. They stimulate the release of and response to insulin. Activation of their receptors by GLP-1 and dual-acting GLP-1/GIP receptor agonists enhance insulin secretion, delay gastric emptying and increase satiety (2). They are approved treatments for obesity in patients with and without diabetes. Tirzepatide, a dual-acting GLP-1/GIP agonist, has shown superior glycaemic control and weight reduction compared with single-agent therapies (3). Initiation typically starts at 2.5 mg once weekly, with dose increments of 2.5 mg every 4 weeks until a target dose (up to 15 mg weekly) is achieved based on tolerability and clinical response. In this case, rapid dose escalation may have contributed to the acute presentation. Tirzepatide’s safety profile is comparable to other incretin-based therapies, with gastrointestinal side effects predominating. In the Tirzepatide Once Weekly for the Treatment of Obesity study, nausea, diarrhoea and constipation were reported by up to 33.3% of participants, leading to discontinuation of the drug in up to 7.1%. However, no cases of ketoacidosis were described (4).

This case also has relevance in the context of post-bariatric surgery care. Metabolic and bariatric surgery is an effective intervention for severe obesity and obesity-associated comorbidities. However, some patients experience insufficient weight loss or subsequent weight regain. Traditionally, revisional bariatric surgery was considered in such cases, although outcomes were often modest and associated with higher morbidity and mortality (5). Adjunctive GLP-1-based pharmacotherapy has emerged as an alternative strategy. A recent study reported that 14% of patients who underwent bariatric surgery between January 2015 and May 2023 were prescribed a GLP-1 or dual-acting GLP-1/GIP receptor agonist during follow-up of up to 10 years (6). Ketosis is common after bariatric surgery due to severe caloric and carbohydrate restriction, often aiding rapid initial weight loss. In contrast, ketoacidosis is rare but has been reported in those with and without diabetes. Ketoacidosis after bariatric surgery is most commonly seen in those with type 1 diabetes, SGLT-2 inhibitor use, poor oral intake, or omission of insulin in the postoperative period. When it does occur, it is usually 1–6 weeks following surgery. The overlap between postoperative gastrointestinal symptoms and those of ketoacidosis can delay recognition (7). To our knowledge, no cases of ketoacidosis have yet been reported with dual-acting GLP-1/GIP receptor agonist use in post-bariatric surgery patients; however, this case highlights a potential risk in this population.

A recent analysis of the FDA Adverse Event Reporting System (FAERS) database examined the association between GLP-1 receptor agonists and ketoacidosis in patients living with diabetes. Over a 5-year period, 1,382 cases of diabetic ketoacidosis and 1,491 cases of ketosis were reported in people receiving GLP-1 receptor agonists. After excluding the impact of SGLT-2 inhibitors and insulin, a disproportionally higher reporting rate of diabetic ketoacidosis persisted. Notably, the higher rate of DKA was not observed when GLP-1 receptor agonists were analysed in combination with insulin, suggesting that relative insulin deficiency may play a central role in the development of this complication (8). Recent case reports highlight the effects of dual-acting tirzepatide therapy, particularly the risk of morbidity associated with developing ketoacidosis. Iqbal *et al.* and Ziyadeh *et al.* also described cases of euglycaemic ketoacidosis in obese patients without diabetes receiving tirzepatide. However, the patients in these cases had no history of prior bariatric surgery. These reports highlight potential contributing factors, such as marked calorie restriction and alcohol intake (1, 9).

We propose that the most likely mechanism underlying the ketoacidosis in our patient was prolonged reduced caloric intake secondary to a gastrointestinal illness, resulting in accelerated ketogenesis. Vomiting, diarrhoea and reduced caloric intake induced by dual-acting GLP-1/GIP receptor agonists can also result in this condition. In addition, dual-acting GLP-1/GIP receptor agonists reduce appetite, slow gastric emptying and may indirectly increase glucagon secretion during stress or fasting, further promoting ketone production (10). Another proposed mechanism is the association between metabolism-related unhealthy obesity and insulin resistance. The patient in this case exhibited features consistent with this phenotype, including visceral fat deposition (hepatic steatosis). Excess free fatty acid deposition in insulin-sensitive non-adipose tissues worsens insulin resistance and can result in relative insulin deficiency (11). Insulin withdrawal or dose reduction has been identified as a factor in the development of DKA among patients treated with GLP-1 receptor agonists (8). Euglycaemic ketoacidosis typically arises when insulin levels are relatively low, but not enough to cause hyperglycaemia, resulting in unchecked lipolysis and ketogenesis. In this case, we hypothesise the suppression of appetite and gastrointestinal upset led to a starvation-like state, reduced insulin action, elevated glucagon levels, and subsequent ketoacidosis despite normal blood glucose. The rarity of this adverse event, coupled with the absence of hyperglycaemia, compounds the diagnostic challenge that can delay recognition and treatment. Clinicians must maintain a high index of suspicion in any patient presenting with metabolic acidosis in the context of GLP-1 or dual-acting agonist therapy.

In conclusion, this case highlights the complex interplay between dual-acting GLP-1/GIP receptor agonists, bariatric surgery and ketoacidosis in patients without diabetes. Prolonged caloric restriction with resultant ketogenesis in the setting of insulin resistance is the most likely mechanism. Increased awareness of this adverse event may aid early recognition and treatment.

## Declaration of interest

The authors declare that there is no conflict of interest that could be perceived as prejudicing the impartiality of the work reported.

## Funding

This research did not receive any specific grant from any funding agency in the public, commercial or not-for-profit sector.

## Patient consent

Written informed consent for publication of their clinical details and/or clinical images was obtained from the patient.

## Author contribution statement

All authors made individual contributions to authorship and were involved in the diagnosis and management of the patient. All authors reviewed and approved the final draft.
